# Supplementary material for: Changes in limiting factors for forager population dynamics in Europe across the last glacial-interglacial transition
Source: Nat Commun. 2022 Sep 6;13:5140. doi: 10.1038/s41467-022-32750-x (PMC9448755; doi:10.1038/s41467-022-32750-x)
Supplement: Supplementary file 1 — Supplementary Information [file 41467_2022_32750_MOESM1_ESM.pdf]

Supplementary Figures

Changes in limiting factors for forager population dynamics in Europe across the Last Glacial-Interglacial Transition

Alejandro Ordonez & Felix Riede

Supplementary figure 1. Estimates of climatically possible mean population density for the 90th percentile (**A**) and 10th percentile (**B**). Estimated average population density for all of Europe is based on the average of grid-based population densities (minimum predicted density for a cell) for the corresponding model in ice-free regions.

Supplementary figure 2. Estimated human population density (persons/100km^2) across Europe for selected times during the 21ky to 8kyBP period. Predictions are based on 90th percentile qGAMs, and any potential human presence is coloured. Areas in grey represent the glacier extent as derived by ICE-6G-C.

Supplementary figure 3. Estimated human population density (persons/100km^2) across Europe for selected times during the 21ky to 8kyBP period. Predictions are based on 10th percentile qGAMs, and any potential human presence is coloured. Areas in grey represent the glacier extent as derived by ICE-6G-C.

Supplementary figure 4. Proportion of the ice-free area of Europe where each variable was estimated to be the factor limiting population density based on 90th percentile (**A**) and 10th percentile (**B**) qGAM models

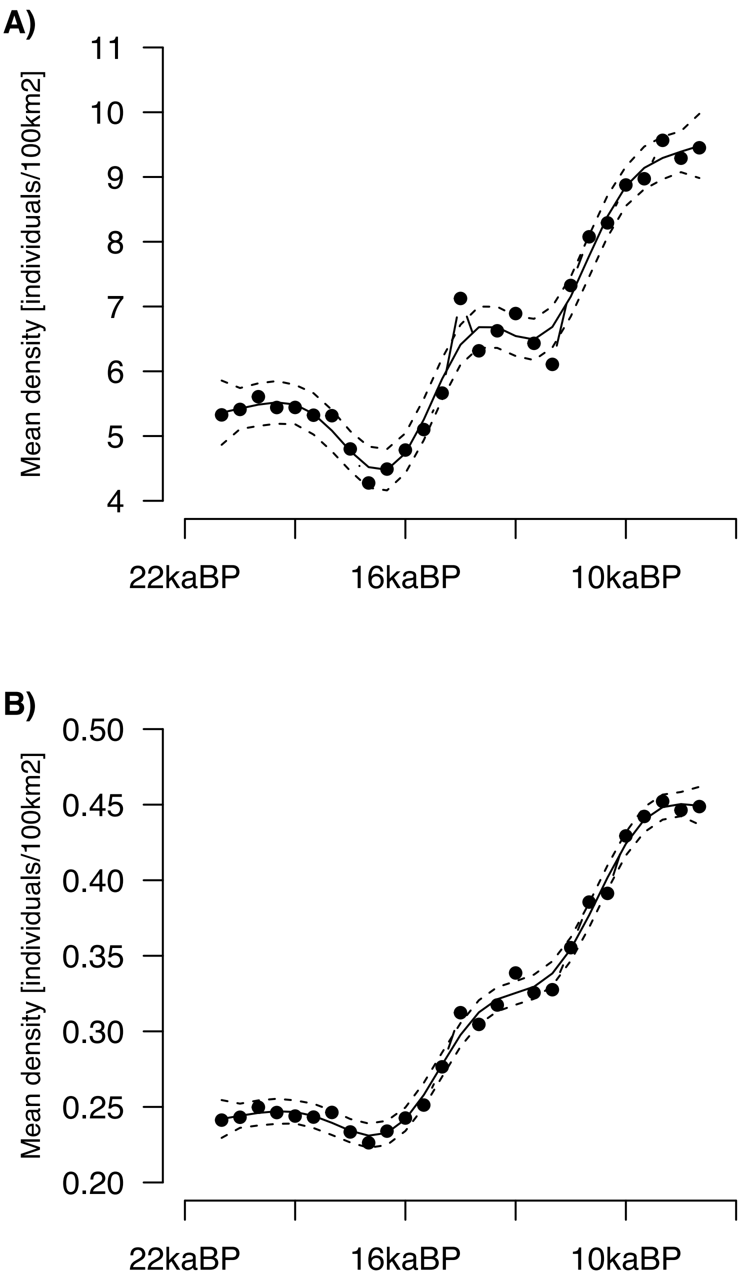

Supplementary figure 1. Estimates of climatically possible mean population density for the 90th percentile (**A**) and 10th percentile (**B**). Estimated average population density for all of Europe is based on the average of grid-based population densities (minimum predicted density for a cell) for the corresponding model in ice-free regions.

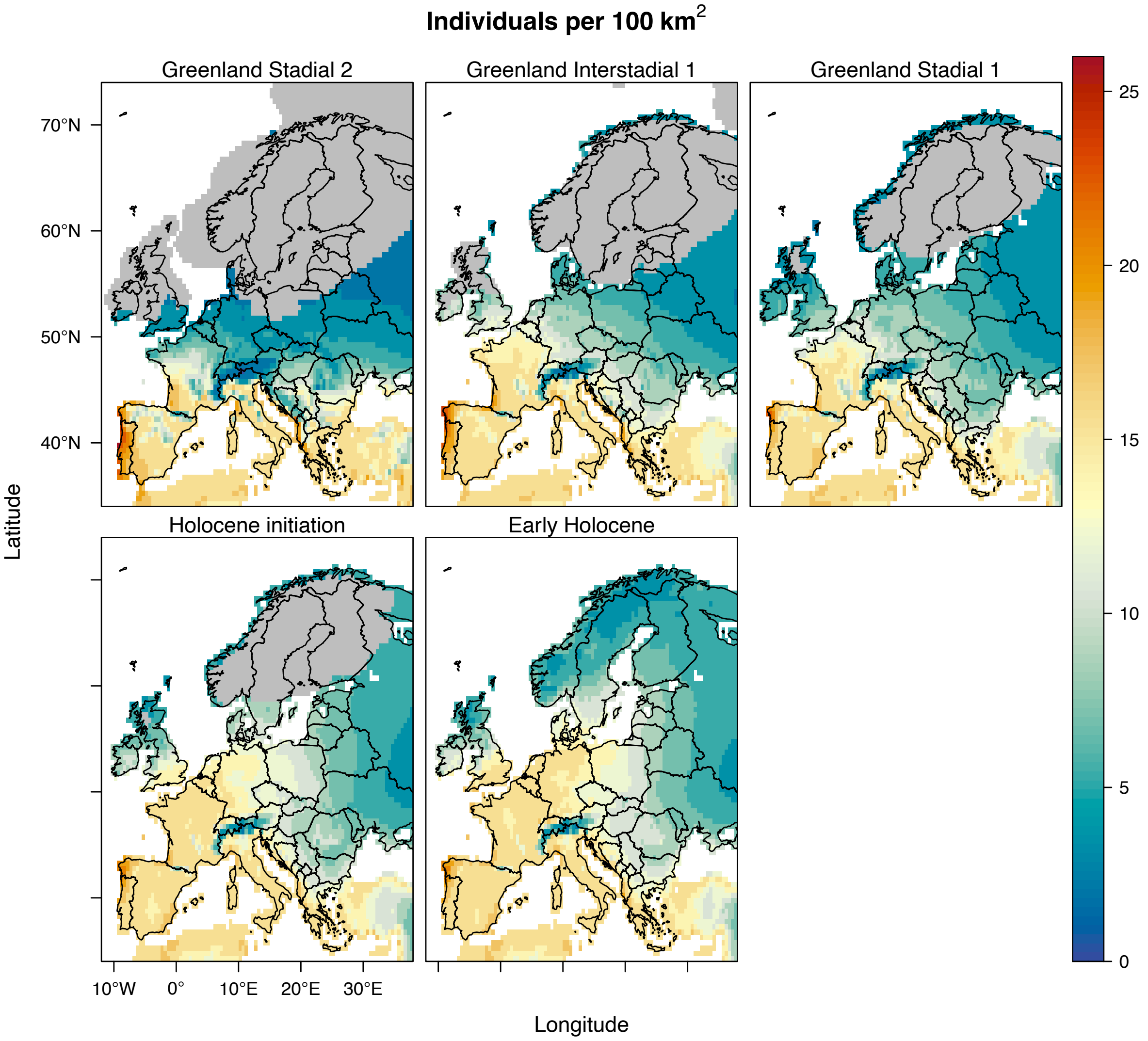

Supplementary figure 2. Estimated human population density (persons/100km^2) across Europe for selected times during the 21ky to 8kyBP period. Predictions are based on 90th percentile qGAMs, and any potential human presence is coloured. Areas in grey represent the glacier extent as derived by ICE-6G-C.

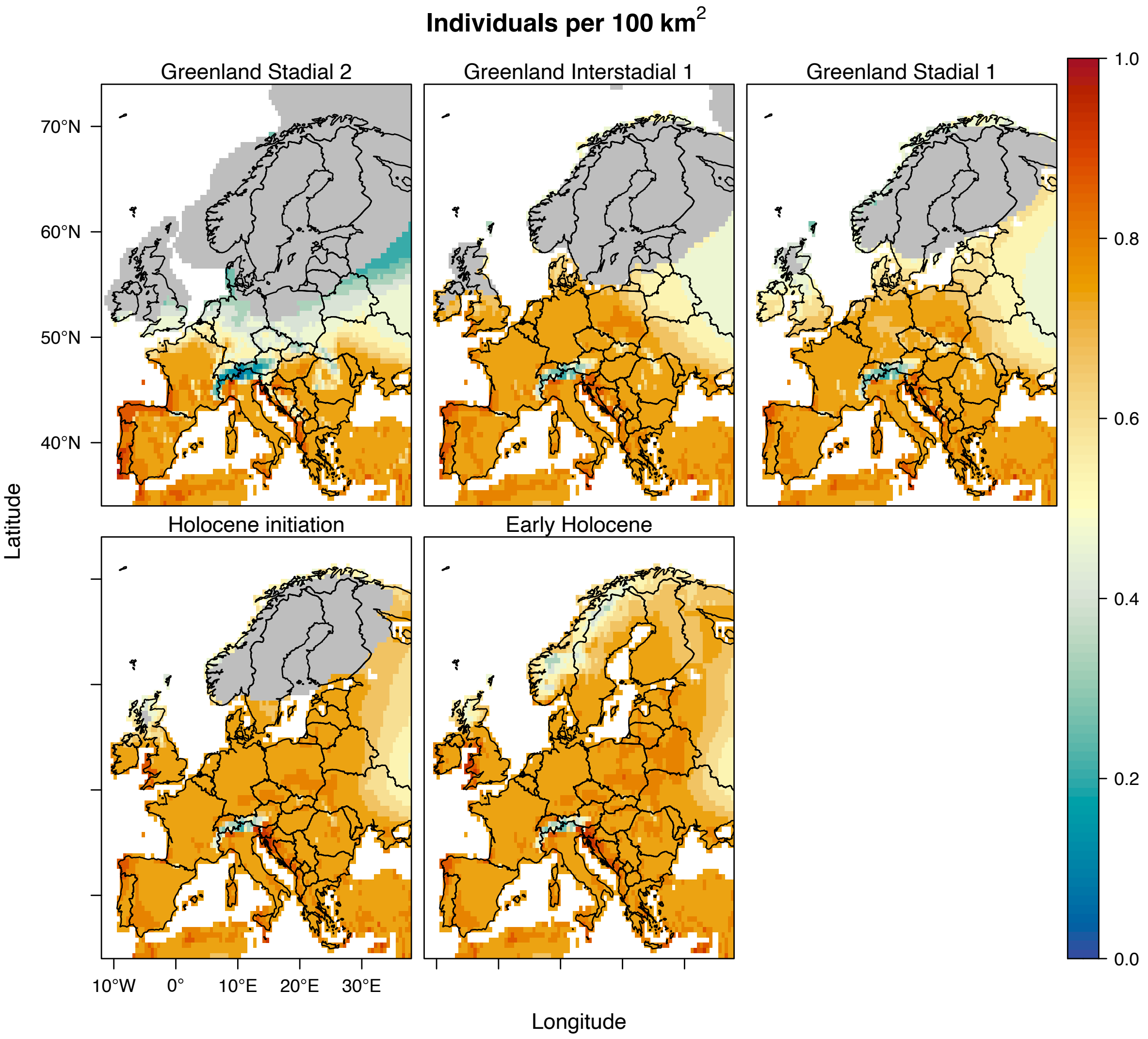

Supplementary figure 3. Estimated human population density (persons/100km^2) across Europe for selected times during the 21ky to 8kyBP period. Predictions are based on 10th percentile qGAMs, and any potential human presence is coloured. Areas in grey represent the glacier extent as derived by ICE-6G-C.

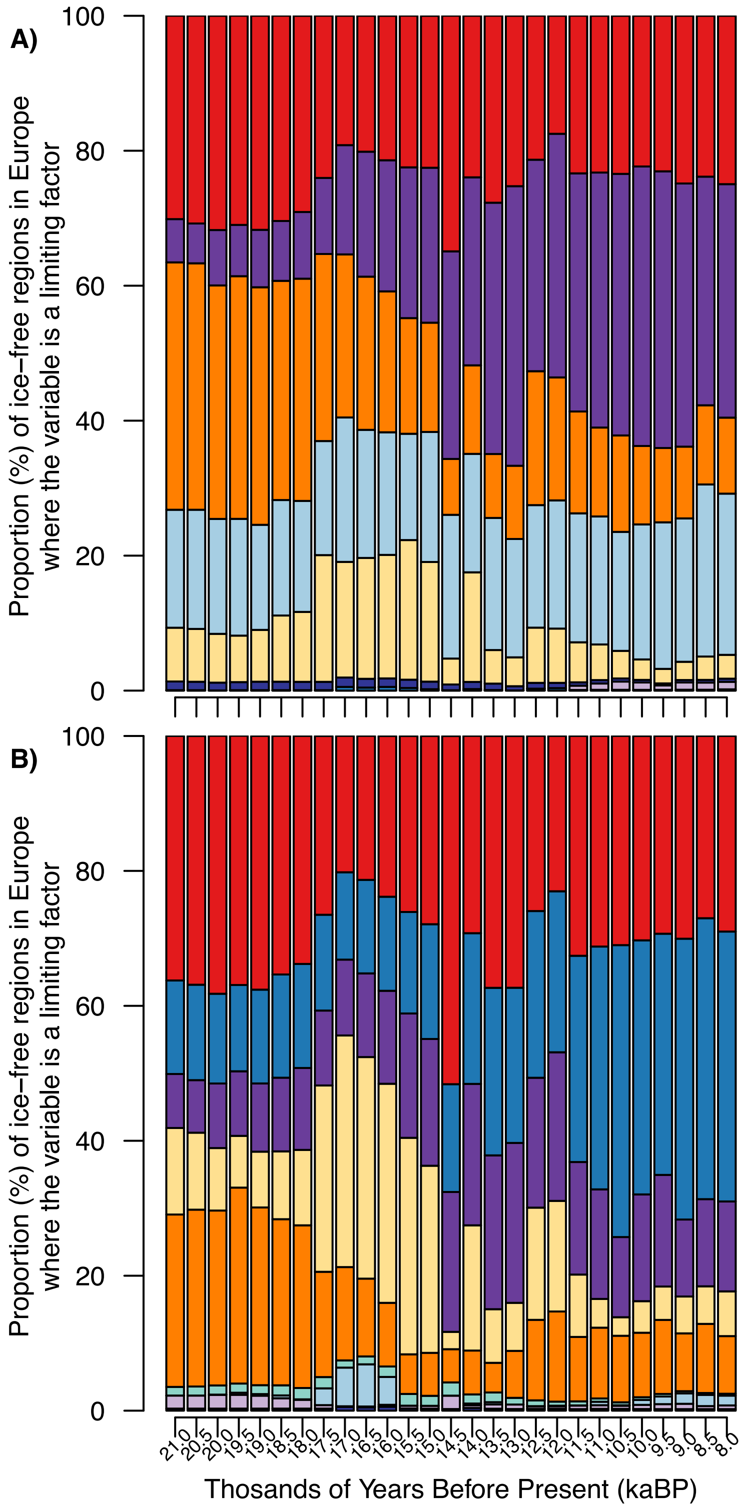

Supplementary figure 4. Proportion of the ice-free area of Europe where each variable was estimated to be the factor limiting population density based on 90th percentile (**A**) and 10th percentile (**B**) qGAM models
