# Supplementary material for: Changes in limiting factors for forager population dynamics in Europe across the last glacial-interglacial transition
Source: Nat Commun. 2022 Sep 6;13:5140. doi: 10.1038/s41467-022-32750-x (PMC9448755; doi:10.1038/s41467-022-32750-x)
Supplement: Supplementary file 3 — Reporting Summary [file 41467_2022_32750_MOESM3_ESM.pdf]

nature portfolio

Corresponding author(s): Alejandro Ordonez

Last updated by author(s): Jul 6, 2022

Reporting Summary

Nature Portfolio wishes to improve the reproducibility of the work that we publish. This form provides structure for consistency and transparency in reporting. For further information on Nature Portfolio policies, see our Editorial Policies and the Editorial Policy Checklist.

Statistics

For all statistical analyses, confirm that the following items are present in the figure legend, table legend, main text, or Methods section.

n/a	Confirmed	
☐	☒	The exact sample size (*n*) for each experimental group/condition, given as a discrete number and unit of measurement
☒	☐	A statement on whether measurements were taken from distinct samples or whether the same sample was measured repeatedly
☒	☐	The statistical test(s) used AND whether they are one- or two-sided<br>*Only common tests should be described solely by name; describe more complex techniques in the Methods section.*
☐	☒	A description of all covariates tested
☐	☒	A description of any assumptions or corrections, such as tests of normality and adjustment for multiple comparisons
☐	☒	A full description of the statistical parameters including central tendency (e.g. means) or other basic estimates (e.g. regression coefficient) AND variation (e.g. standard deviation) or associated estimates of uncertainty (e.g. confidence intervals)
☒	☐	For null hypothesis testing, the test statistic (e.g. *F*, *t*, *r*) with confidence intervals, effect sizes, degrees of freedom and *P* value noted<br>*Give P values as exact values whenever suitable.*
☒	☐	For Bayesian analysis, information on the choice of priors and Markov chain Monte Carlo settings
☒	☐	For hierarchical and complex designs, identification of the appropriate level for tests and full reporting of outcomes
☒	☐	Estimates of effect sizes (e.g. Cohen's *d*, Pearson's *r*), indicating how they were calculated

Our web collection on statistics for biologists contains articles on many of the points above.

Software and code

Policy information about availability of computer code

Data collection	No software was used for data collection,
Data analysis	All analyses were conducted using R V. 3.6 using the packages mgcv (version 1.8.24;99) and qgam (version 1.3.2;100) for the (quantile) Generalized Additive Models; raster (V. 3.4-13) and maptools (V. 1.1-2) for creating, manipulating, and visualizing Spatial objects; and Base R for the manipulation, summarization and graphical representation of the results. Code and subset of the data used in this study is available at https://doi.org/10.5281/zenodo.6962693.

For manuscripts utilizing custom algorithms or software that are central to the research but not yet described in published literature, software must be made available to editors and reviewers. We strongly encourage code deposition in a community repository (e.g. GitHub). See the Nature Portfolio guidelines for submitting code & software for further information.

Data

Policy information about availability of data

All manuscripts must include a data availability statement. This statement should provide the following information, where applicable:

- Accession codes, unique identifiers, or web links for publicly available datasets
- A description of any restrictions on data availability
- For clinical datasets or third party data, please ensure that the statement adheres to our policy

The 'Binford' ethnographic database is available from the Database of Places, Language, Culture, and Environment (D-PLACE; https://d-place.org/about). Current and Late Quaternary environmental datasets are publicly available from the associated references. International Union for Quaternary Science (INQUA) Radiocarbon Palaeolithic Europe Database v28 is available from https://pandoradata.earth/dataset/radiocarbon-palaeolithic-europe-database-v28. Contemporary climate databases are available from the WorldClim V.2.1. project (https://www.worldclim.org/data/worldclim21.html), and Late Pleistocene climate sources are available

at https://doi.org/10.6084/m9.figshare.c.4673120.v2.

Field-specific reporting

Please select the one below that is the best fit for your research. If you are not sure, read the appropriate sections before making your selection.

☐ Life sciences ☐ Behavioural & social sciences ☒ Ecological, evolutionary & environmental sciences

For a reference copy of the document with all sections, see nature.com/documents/nr-reporting-summary-flat.pdf

Ecological, evolutionary & environmental sciences study design

All studies must disclose on these points even when the disclosure is negative.

Study description	Here we evaluated changes in hunter-gatherers palaeodemography in Europe from the Last Glacial Maximum (Greenland Stadial 2, GS2) to 8000 years before present (BP). We begin with the global ethnographic hunter-gatherer dataset originally assembled by Binford. However, we used a new conceptual approach to evaluate spatiotemporal dynamics of hunter-gatherers' demographic trends – the limiting factor idea. We modelled the changes in population density as a function of 16 environmental variables and increased the model into climatic conditions for the 21kaBP to 8kaBP period. By taking this approach, we were able to determine, within a hypothesis-driven framework, how specific limiting factors acted on forager populations and how these limiting factors varied over space and time.
Research sample	We use a selection of 159 populations form those included in the original 'Binford' ethnographic database that is available on line. This selection removed any "subsidies" to the hunter-gatherers lifestyle, hence represents those with a fully hunter-gatherers.
Sampling strategy	Starting with the full original 'Binford' ethnographic database we removed: 1) Observations associated with food producers; sedentary populations or those that reside at a single location for >1 year; 2) populations using aquatic resources (>30% of their dietary protein comes from aquatic environments); and 3) observations related to horse-riding populations. We generated our paleoclimatic variables by aggregating the original decadal averages of monthly values to centennial. The original climate data source is a bias-corrected and downscaled paleoclimatic surface at a 30-ArcMin resolution.
Data collection	The lead author downloaded all datasets from on-line public repositories.
Timing and spatial scale	Th datasets were downloaded on the 1st of December 2021
Data exclusions	All data from our subsample of 'Binford' ethnographic database were used.
Reproducibility	N/A as this is a database study
Randomization	N/A as this is a database study
Blinding	N/A as this is a database study

Did the study involve field work? ☐ Yes ☒ No

Reporting for specific materials, systems and methods

We require information from authors about some types of materials, experimental systems and methods used in many studies. Here, indicate whether each material, system or method listed is relevant to your study. If you are not sure if a list item applies to your research, read the appropriate section before selecting a response.

Materials & experimental systems

n/a	Involved in the study
☒	☐ Antibodies
☒	☐ Eukaryotic cell lines
☒	☐ Palaeontology and archaeology
☒	☐ Animals and other organisms
☒	☐ Human research participants
☒	☐ Clinical data
☒	☐ Dual use research of concern

Methods

n/a	Involved in the study
☒	☐ ChIP-seq
☒	☐ Flow cytometry
☒	☐ MRI-based neuroimaging
